# Latent profiles of moral sensitivity and its associated factors among operating room nurses

**DOI:** 10.3389/fmed.2026.1889711

**Published:** 2026-08-14

**Authors:** Xiu-yun Chen, Fang Yan, Yi Liu, Li-jun He, Chao-li Xue, Rong-hua Gong

**Affiliations:** 1 Department of Operating Room, Northern Jiangsu People's Hospital, Yangzhou, Jiangsu, China; 2 Department of Nursing, Northern Jiangsu People's Hospital, Yangzhou, Jiangsu, China

**Keywords:** influencing factors, latent profile analysis, moral sensitivity, operating room nurses, role clarity

## Abstract

**Objective:**

To identify latent profiles of moral sensitivity among operating room nurses and to examine factors influencing these profiles.

**Methods:**

A cross-sectional study was conducted from May to July 2025. A total of 579 operating room nurses from 18 tertiary hospitals in Jiangsu Province, China, were recruited using convenience sampling. Data were collected using the Chinese version of the Moral Sensitivity Questionnaire-Revised Version (MSQ-R-CV), the Nurse Work Environment Perception Questionnaire, and the Role Clarity Scale. Latent profile analysis (LPA) was performed using Mplus 8.0. Binary logistic regression was conducted using SPSS 26.0 to identify independently associated factors of latent profile membership.

**Results:**

Two distinct latent profiles were identified: a low moral sensitivity group (42.8%) and a high moral sensitivity group (57.2%). Binary logistic regression revealed that gender (male vs. female: OR = 44.27, 95% CI: 5.34–366.93, *P* < 0.001), age (≥45 years vs. < 25 years: OR = 9.33, 95% CI: 3.87–22.51, *P* < 0.001), years of experience (5–10 years vs. 1–5 years: OR = 22.64, 95% CI: 10.53–48.70, *P* < 0.001), and role clarity (OR = 1.09, 95% CI: 1.03–1.14, *P* < 0.001) were significant independently associated factors of high moral sensitivity.

**Conclusion:**

The identification of a substantial low-sensitivity subgroup underscores the urgent need for targeted ethics training, particularly for younger and less experienced female nurses. Future targeted interventions focusing on role clarification, particularly for younger, less experienced, and female nurses, may effectively enhance moral sensitivity in this population.

## 1 Introduction

With the transformation of healthcare delivery models and the convergence of diverse values in multicultural contexts, operating room (OR) nurses frequently encounter complex ethical dilemmas in clinical practice. Unlike other clinical settings, the OR is characterized by high time pressure, technology-driven workflows, and patients who are often under general anesthesia—rendering them unable to participate in decision-making or voice concerns (1, 2). These unique conditions often involve sensitive ethical issues, such as end-of-life care decisions, the implementation of informed consent, and the protection of patient privacy.

Moral sensitivity is defined as the ability to recognize and interpret ethical issues in clinical practice. It is regarded as a fundamental component of ethical behavior (3) and a critical foundation for ethical decision-making capability (4). For nurses, moral sensitivity refers to the capacity to maintain acute awareness of moral values implicated in ethical conflicts within clinical practice settings and to possess conscious recognition of the responsibilities assumed in one's professional role. Elevated moral sensitivity enhances nurses' empathy, strengthens moral care awareness, improves job satisfaction, and promotes the quality of ethical decision-making (5, 6).

Previous studies on nurses' moral sensitivity have primarily focused on intensive care units, emergency departments, palliative care wards, pediatrics, psychiatry, and infectious disease departments (7), with limited attention to the OR setting. A growing body of evidence has identified various factors influencing moral sensitivity among nurses, including demographic characteristics (e.g., age, gender, education level, years of experience), work environment factors, and individual psychological attributes (8–10). Recent studies have also highlighted the role of organizational factors, such as ethical climate and leadership support, in shaping nurses' moral sensitivity (11, 12). However, most of these studies have adopted a variable-centered approach, which assumes that the study population is homogeneous with respect to the relationships among variables, potentially masking important subgroup differences.

Latent profile analysis (LPA) is a person-centered statistical method that classifies individuals into latent subgroups based on their response patterns to observed variables. Unlike traditional variable-centered approaches (e.g., regression), LPA allows for the identification of distinct subpopulations with shared characteristics, enabling in-depth investigation of heterogeneity among different groups in terms of demographic characteristics, clinical manifestations, and influencing factors, thus providing a basis for precision interventions (13). However, LPA has not been applied to examine the heterogeneity of moral sensitivity among OR nurses. A systematic literature search in PubMed, Web of Science, and CNKI databases (up to Feburary 2026) using keywords “moral sensitivity,” “latent profile analysis,” and “operating room nurses” yielded no relevant studies. While LPA has been increasingly applied in nursing research to identify distinct profiles of burnout, compassion fatigue, and professional identity (14, 15), its application to moral sensitivity among OR nurses remains unexplored.

Therefore, this study aims to: (1) identify distinct latent profiles of moral sensitivity among Chinese OR nurses using LPA, and (2) examine the demographic and occupational factors (including work environment perception and role clarity) that are associated with these profiles. The findings will provide empirical evidence for nursing administrators to develop targeted, profile-specific intervention strategies tailored to groups with distinct moral sensitivity profiles.

## 2 Methods

### 2.1 Study design and participants

A cross-sectional study was conducted from May to July 2025. Using convenience sampling, 579 operating room nurses from 18 tertiary hospitals in Jiangsu Province, China, were recruited as participants. Inclusion criteria were: (a) currently employed, having passed the nurse license examination and being registered; (b) having at least 1 year of operating room work experience; and (c) providing informed consent and voluntarily participating in the study. Exclusion criteria were: (a) nurses who were interns, undergoing further training from other hospitals, or on leave; and (b) those who declined to participate.

According to sample size estimation principles for cross-sectional studies (9), the sample size should be 10 to 15 times the number of independent variables. This study included 11 independent variables, requiring a sample size of at least 110 to 165. Considering a 20% attrition rate, the required sample size was increased to 163 to 198. For latent profile analysis, simulation studies suggest that a sample size of at least 500 is recommended to ensure stable class enumeration and adequate power (16, 17). Ultimately, 579 participants were recruited using convenience sampling, which exceeds both the regression-based and LPA-specific recommendations. A *post-hoc* power analysis indicated that this sample size was sufficient to detect medium effects (power > 0.80) for all analyses conducted. All participants provided written informed consent. The study was approved by the Ethics Committee of Northern Jiangsu People's Hospital (Ethics Approval Number: 2022ky088).

### 2.2 Data collection instruments

#### 2.2.1 General information questionnaire

A self-developed general information questionnaire was administered, which included gender, age, marital status, highest educational attainment, professional title, years of work experience, employment type, and average weekly working hours.

#### 2.2.2 Chinese version of the moral sensitivity questionnaire-revised version (MSQ-R-CV)

We employed the MSQ-R-CV translated and culturally adapted by Huang et al. (10). The scale consists of two dimensions and nine items: moral responsibility and strength (five items) and moral burden (four items). A six-point Likert scale was used, with one indicating “strongly disagree” and six indicating “strongly agree,” resulting in a score range of nine to 54. Higher scores indicate greater moral sensitivity. The Cronbach's α coefficient in this study was 0.852, indicating good internal consistency.

#### 2.2.3 Nurse work environment perception questionnaire (WEQ)

The WEQ was developed by Blegen et al. (13) to assess nurses' perceptions of the hospital work environment. The instrument was translated and revised into Chinese by Jiang et al. (14). The revised questionnaire comprises 19 items across four dimensions: medical resource allocation, quality management, human resources, and colleague relationships. A five-point Likert scale was employed, with total scores ranging from 19 to 95; higher scores indicate a higher level of nurse approval of the work environment. The Cronbach's α coefficient in this study was 0.864, indicating good reliability.

#### 2.2.4 Role clarity scale (RCS)

The RCS, developed by Rizzo et al. (15) and translated by Zhang et al. (18), consists of a unidimensional set of six items. Scoring was performed using a seven-point Likert scale, where one corresponds to “strongly disagree” and seven to “strongly agree.” The total score ranges from six to 42, with higher scores indicating greater role clarity among healthcare personnel. The Cronbach's α coefficient in this study was 0.802, indicating good reliability.

### 2.3 Survey procedures

This study used the Questionnaire Star platform (www.wjx.com) to construct electronic questionnaires, comprising two sections: informed consent and the formal survey. Following approval from the nursing management departments of the participating hospitals, the respective departments uniformly distributed the questionnaire link to the study participants. The survey used standardized instructions to clarify the completion requirements. Participants were required to read the informed consent information and select the “Agree” option before accessing the formal questionnaire section. All items were mandatory and had to be fully completed before submission.

During data collection, quality control was conducted through a combination of automatic logical verification via the Questionnaire Star system and manual verification by researchers. A total of 586 questionnaires were distributed. Following the exclusion of seven invalid questionnaires (completion time under 100 s, determined based on a pilot test of 20 nurses as three standard deviations below the mean completion time), 579 valid questionnaires were retained, yielding an effective response rate of 98.81%.

### 2.4 Statistical analysis

Latent profile analysis (LPA) of operating room nurse moral sensitivity was performed using Mplus 8.0 software (Muthén & Muthén, Los Angeles, CA, USA),, using the nine items of the MSQ-R-CV as manifest variables. LPA models with one to five classes were sequentially fitted. Model fit was assessed using the Akaike Information Criterion (AIC), Bayesian Information Criterion (BIC), and adjusted Bayesian Information Criterion (aBIC), with lower values indicating better model fit. Entropy values closer to 1 reflect greater classification accuracy. The Lo-Mendell-Rubin adjusted likelihood ratio test (LMRT) and the Bootstrap Likelihood Ratio Test (BLRT) were used to compare models with *k* vs. *k*-1 classes, with a statistically significant result (*P* < 0.05) indicating that the *k*-class model was superior. The optimal model also had to meet the requirement that the minimum class sample size was no less than 5% of the total sample size.

Before conducting binary logistic regression, multicollinearity among independent variables was assessed using the variance inflation factor (VIF). A VIF > 5 was considered indicative of significant multicollinearity. All VIF values were below two, indicating no substantial multicollinearity.

Other statistical analyses were performed using SPSS version 26.0 (IBM Corp., Armonk, NY, USA). Measurement data conforming to a normal distribution are presented as mean ± standard deviation, whereas categorical data are expressed as frequency and percentage. Univariate analyses were conducted using chi-square tests and independent-sample *t*-tests, while multivariate analysis was performed using binary logistic regression to identify independent influencing factors. The significance threshold was set at *P* < 0.05. There were no missing data in the final analytical sample, as all questionnaire items were mandatory and required full completion before submission.

## 3 Results

### 3.1 Latent profile analysis results of moral sensitivity among operating room nurses

The moral sensitivity score of operating room nurses was 38.216 ± 0.59. This study utilized Mplus 8.0 to conduct LPA, employing the nine items of the MSQ-R-CV as manifest variables, and sequentially constructed latent profile models ranging from one to five classes. As the number of fitted classes increased from one to five, the AIC, BIC, and aBIC values progressively decreased, indicating improved model fit. The 2-class model demonstrated high classification accuracy (Entropy = 0.953) and a significant LMR test (*P* < 0.001). Although the 3-class and 4-class models had lower AIC, BIC, and aBIC values, each produced at least one class with a proportion of less than 5% of the total sample, suggesting potentially unstable or non-replicable subgroups. The 5-class model yielded a non-significant LMR test (*P* = 0.064). Following established criteria for LPA model selection (16)—including lower AIC/BIC/aBIC, Entropy > 0.80, significant LMR (*P* < 0.05), and class proportions > 5%—the 2-class model was selected as the optimal solution (Table 1).

**Table 1:** Model fit indices for latent profile models of moral sensitivity among operating room nurses (*n* = 579).

Model type	AIC	BIC	aBIC	Entropy	LMR (*P*)	BLRT (*P*)	Class probability
1	15,189.701	15,268.204	15,211.061	–	–	–	–
2	13,716.465	13,838.581	13,749.692	0.953	<0.001	<0.001	0.428/0.572
3	13,121.951	13,287.681	13,167.046	0.976	<0.001	<0.001	0.021/0.409/0.570
4	12,610.719	12,820.061	12,667.680	0.985	<0.001	<0.001	0.021/0.051/0.355/0.573
5	12,002.863	12,255.819	12,071.692	0.968	0.0639	0.0778	0.050/0.021/0.325/0.187/0.418

AIC, akaike information criterion; BIC, Bayesian Information Criterion; aBIC, adjusted Bayesian Information Criterion; LMR, Lo-Mendell-Rubin adjusted likelihood ratio test; BLRT, bootstrap likelihood ratio test.

**Figure 1:**
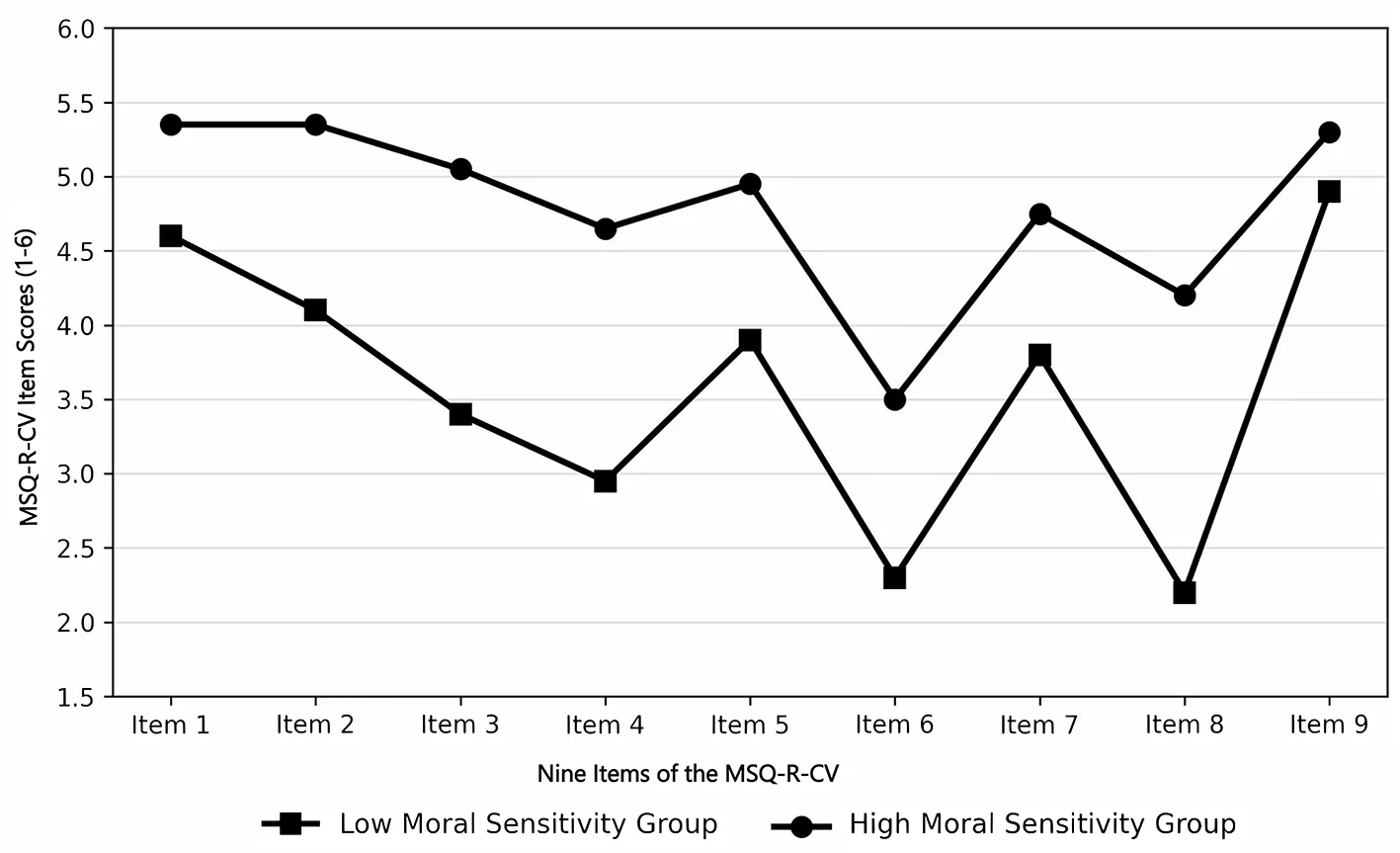
Latent profile analysis of moral sensitivity among operating room nurses. The y-axis represents item scores (range: 1–6), and the x-axis represents the nine items of the MSQ-R-CV.

The results indicate that the average membership probabilities for the two latent profiles were 0.986 and 0.994, respectively, indicating good reliability of the model classification results, as detailed in Table 2. Based on the characteristics of the latent classes, Class 1 scored generally lower across items and was designated the “Low moral sensitivity group,” accounting for 42.8%. Class 2 exhibited higher scores across all items and was designated as the “High Moral Sensitivity Group,” comprising 57.2% of the sample, as illustrated in Figure 1.

**Table 2:** Latent profile membership probabilities for two moral sensitivity classes among operating room nurses (*n* = 579, %).

Latent Profile	Low moral sensitivity group	High moral sensitivity group
Low moral sensitivity group	0.986	0.014
High moral sensitivity group	0.006	0.994

### 3.2 Univariate analysis of latent profiles of moral sensitivity among operating room nurses

Latent profiles of moral sensitivity among Operating Room Nurses were grouped to compare differences in demographic characteristics, nurse work environment perception, and role clarity. Univariate analysis results indicated statistically significant differences in the distribution of general demographic characteristics, including gender, professional title, and years of service (*P* < 0.05), as shown in Table 3. Significant differences were observed in Nurse Work Environment Perception and Role Clarity scores between the two latent profiles (*P* < 0.05), as presented in Table 4.

**Table 3:** Comparison of general demographic characteristics of operating room nurses across different latent profiles (*n*, %).

Item	Number of cases (*n*)	Low moral sensitivity group (*n* = 248)	High moral sensitivity group *(n* = 331)	*χ^2^*	*P value*
Gender					
Male	44	1 (0.4)	43 (13.0)	31.992	<0.001
Female	535	247 (99.6)	288 (87.0)		
Age (years)					
<25	110	86 (34.7)	24 (7.3)	84.537	<0.001
25–34	261	107 (43.2)	154 (46.5)		
35–44	133	43 (17.3)	90 (27.2)		
>45	76	12 (4.8)	63 (19.0)		
Marital status					
Unmarried	152	64 (25.8)	88 (26.6)	0.323	0.956
Married without children	20	9 (3.6)	11 (3.3)		
Married with children	395	169 (68.2)	226 (68.3)		
Divorced	12	6 (2.4)	6 (1.8)		
Highest educational qualification					
Master's degree	35	18 (7.3)	17 (5.1)	1.366	0.505
Bachelor's degree	483	206 (83.0)	277 (83.7)		
Associate degree	60	24 (9.7)	37 (11.2)		
Professional title					
Nurse	43	21 (8.5)	22 (6.6)	7.235	0.124
Nurse practitioner	171	71 (28.6)	100 (30.2)		
Supervising nurse practitioner	299	132 (53.2)	167 (50.5)		
Deputy chief nurse practitioner	40	19 (7.7)	21 (6.3)		
Chief nurse	26	5 (2.0)	21 (6.3)		
Years of service					
1–5 years	127	116 (46.8)	11 (3.3)	157.119	<0.001
5–10 years	194	52 (21.0)	142 (42.9)		
>10 years	258	80 (32.2)	178 (53.8)		
Employment type					
Contract	489	209 (84.3)	280 (84.6)	0.011	0.927
Established Post	90	39 (15.7)	51 (15.4)		
Average weekly working hours					
<50	142	52 (21.0)	90 (27.2)	4.196	0.123
50–60	298	139 (56.0)	159 (48.0)		
>60	139	57 (23.0)	82 (24.8)		

**Table 4:** Comparison of nurse work environment perception and role clarity among operating room nurses with different latent profiles.

Item	Overall (*n* = 579)	Latent profile category		*t*	*P*
		Low moral sensitivity group	High moral sensitivity group		
Nurse work environment perception	74.571 ± 0.10	73.211 ± 1.30	75.598 ± 0.99	−2.816	0.003
Role clarity	35.365 ± 0.08	34.005 ± 0.65	36.394 ± 0.35	−5.743	<0.001

### 3.3 Binary logistic regression results of latent profiles of moral sensitivity among operating room nurses

Using the two categories of operating room nurse moral sensitivity identified by LPA as the dependent variable (Low moral sensitivity group = 0, High Moral Sensitivity Group = 1), five variables with statistically significant differences in univariate analysis (gender, age, years of service, nurse work environment perception, and role clarity scores) were included as independent variables in binary logistic regression analysis. The results indicate that gender, age, and role clarity scores are significant associated factors of the high moral sensitivity latent profiles of operating room nurses. Specifically, male nurses, those of greater age, longer years of service, and higher role clarity scores exhibit higher levels of moral sensitivity; details are shown in Table 5.

**Table 5:** Binary logistic regression analysis of latent profiles of operating room nurse moral sensitivity.

Variable	β	SE	Wald *χ^2^*	*P*	OR	95% CI
Constant term	−6.984	1.138	37.69	<0.001	0.001	
Gender (Reference: female)						
Male	3.79	1.079	12.34	<0.001	44.272	(5.342, 366.93)
Age (Reference:<25)						
25–34	0.768	0.322	5.687	0.017	2.155	(1.146, 4.049)
35–44	1.275	0.364	12.258	<0.001	3.578	(1.753,7.305)
>45	2.233	0.449	24.73	<0.001	9.332	(3.87, 22.506)
Years of service (Reference: 1–5 years)						
5–10	3.12	0.391	63.733	<0.001	22.639	(10.525, 48.695)
>10	2.624	0.377	48.323	<0.001	13.788	(6.58, 28.893)
Role clarity score	0.082	0.025	10.534	<0.001	1.085	(1.033, 1.14)

The odds ratio for gender (male vs. female) is notably high with a wide confidence interval, likely due to the small proportion of male nurses (7.6%) and the high prevalence of high moral sensitivity within this subgroup (97.7%). This estimate should be interpreted with caution. The logistic regression model showed adequate fit to the data. The Hosmer–Lemeshow test yielded a non-significant result (χ^2^ = 8.473, df = 8, *P* = 0.389), indicating no evidence of poor model fit. The Nagelkerke *R*^2^ was 0.742, suggesting that the model explained a substantial proportion of the variance in profile membership. The overall model was statistically significant (omnibus χ^2^ = 327.614, df = 8, *P* < 0.001), with a classification accuracy of 91.2%.

## 4 Discussion

### 4.1 Major findings

This study is the first to apply latent profile analysis (LPA) to identify distinct subgroups of moral sensitivity among operating room nurses in China. Our findings revealed two distinct profiles: a low moral sensitivity group (42.8%) and a high moral sensitivity group (57.2%). The overall moral sensitivity score was 38.21 ± 6.59, indicating a moderate level. Binary logistic regression identified gender, age, years of service, and role clarity as significant independently associated factors of profile membership. Notably, nurses in the 5–10 year range showed the highest odds of belonging to the high sensitivity group. These findings have important implications for developing targeted interventions to enhance moral sensitivity among OR nurses.

### 4.2 Current status and profiles of moral sensitivity among OR nurses

The mean moral sensitivity score in our sample (38.21 ± 6.59) represents a moderate level, which is consistent with a previous study conducted in pediatric settings (19) but slightly lower than reports among psychiatric nurses (20). This moderate level may reflect the unique characteristics of the OR environment. Unlike other clinical settings where nurses have direct and continuous interaction with conscious patients, OR nurses primarily focus on surgical procedures, instrument handling, and intraoperative patient monitoring. In this task-centered, technology-driven environment, ethical considerations may be inadvertently overshadowed by technical and procedural demands.

Importantly, our LPA revealed that 42.8% of OR nurses belonged to the low moral sensitivity group, indicating that a substantial proportion of nurses exhibit lower sensitivity levels. This finding is concerning given that moral sensitivity is foundational to ethical decision-making and patient-centered care (3). As Milch and Pedersen (21) noted, the time pressures and efficiency-driven nature of the OR may cause nurses to perceive moral concerns as “secondary issues,” leading to moral disengagement or neglect of ethical issues. This is particularly problematic in the OR setting, where patients are often under general anesthesia and entirely unable to autonomously express or protect their rights. Therefore, enhancing moral sensitivity among OR nurses is not merely an educational priority but a patient safety imperative.

### 4.3 Factors associated with profiles of moral sensitivity

#### 4.3.1 Gender

Our results indicate a very strong association between being male and belonging to the high moral sensitivity group (OR = 44.27). While this may reflect genuine differences in empathic patterns under high-pressure conditions, the magnitude of this effect is likely inflated by the small number and skewed distribution of male nurses in our sample. Male OR nurses had significantly higher odds of belonging to the high moral sensitivity group compared to their female counterparts. This finding contrasts with some previous studies that reported no gender differences in moral sensitivity (8, 9). One possible explanation is that in high-pressure OR environments, female nurses may experience heightened empathetic involvement, increasing their vulnerability to compassion fatigue. As a psychological self-protective response, their moral sensitivity may be temporarily diminished (22). Conversely, male nurses may exhibit different patterns of empathy and emotional regulation, resulting in relatively slower “depletion” of moral sensitivity throughout their careers. However, given the wide confidence interval for this estimate (likely due to the small proportion of male nurses in our sample, *n* = 44, 7.6%), this finding should be interpreted with caution and warrants replication in larger, more gender-balanced samples. Despite the statistical significance, the extreme OR value (44.27) with an extremely wide confidence interval (5.34–366.93) should be interpreted as a provisional observation rather than a precise effect size. The small number of male participants (*n* = 44, 7.6%) and the near-ceiling prevalence of high moral sensitivity within this subgroup (43 out of 44, 97.7%) likely contributed to this statistical artifact. Therefore, this finding should be considered hypothesis-generating rather than conclusive, and it underscores the urgent need for future studies with larger and more gender-balanced samples to reliably estimate gender differences in moral sensitivity.

#### 4.3.2 Age and years of service

Consistent with previous studies (23–25), we found that older nurses and those with longer years of service were more likely to belong to the high moral sensitivity group. This can be explained by the continuous development of moral sensitivity through interactions between individuals and their social environment. As Rest (26) demonstrated, the accumulation of age and work experience enhances the ability to discern complex situations, facilitating more accurate identification of ethical transgressions. With increasing clinical experience, OR nurses accumulate substantial exposure to ethical decision-making scenarios, enabling deeper comprehension of clinical complexity and more rapid identification of ethical conflicts.

However, a particularly noteworthy finding emerged from our regression analysis: nurses with 5–10 years of experience had the highest odds of belonging to the high moral sensitivity group, followed by those with >10 years. This observed pattern—where moral sensitivity appeared to peak at 5–10 years and then slightly declined—has not been previously reported. While this suggests a potential nonlinear trend, formal tests of nonlinearity were not conducted; therefore, this observation should be interpreted as an exploratory finding that warrants further investigation in future studies. One possible explanation is that nurses at the 5–10 year career stage often assume greater clinical responsibilities, such as precepting new staff or coordinating complex surgeries, which may heighten their awareness of ethical issues. In contrast, nurses with >10 years of experience may experience moral residue, compassion fatigue, or emotional numbing as a cumulative result of prolonged exposure to ethical conflicts, potentially blunting their moral sensitivity over time (27). This finding suggests that interventions to sustain moral sensitivity should target not only novice nurses but also those in later career stages who may be at risk of moral desensitization.

#### 4.3.3 Role clarity

Role clarity emerged as a significant independently associated factor of moral sensitivity. Role clarity refers to the extent to which healthcare personnel clearly understand their job responsibilities, role definitions, and organizational strategic goals. A high degree of role clarity effectively stimulates intrinsic work motivation, facilitates the realization of personal professional values, and enhances both professional identity and the sense of dignified work (28, 29). According to social identity theory, strengthening professional identity can significantly bolster practitioners' emotional connections, sense of self-worth, and confidence in their abilities, thereby increasing their intrinsic motivation to achieve professional goals. Building on this theory, a well-defined professional identity promotes greater work engagement, encouraging nurses to proactively enhance their ethical literacy and practical experience, which in turn substantially improves their moral decision-making capabilities (30, 31). Moreover, when individuals possess a clear understanding of their roles, responsibilities, and objectives, they are better able to perceive others' needs and expectations, allowing for more accurate identification of patients' clinical needs and ethical dilemmas. This finding has important practical implications, as role clarity is a modifiable organizational factor that can be enhanced through structured orientation programs, clear job descriptions, and regular performance feedback.

### 4.4 Implications for nursing management

Based on our findings, we propose targeted, profile-specific intervention strategies for OR nurses. For the low moral sensitivity group—which comprised more female, younger, and less experienced nurses—nursing managers should implement structured, simulation-based ethics training programs. These programs should focus on recognizing ethical dilemmas in common OR scenarios, such as intraoperative patient positioning for privacy, handling of retained surgical items, informed consent for unconscious patients, and end-of-life decision-making. Case-based learning, reflective practice exercises, and team-based ethical discussions may be particularly effective for this group. Given that role clarity was a significant predictor, managers should also ensure that novice nurses receive clear job descriptions and regular feedback to enhance their understanding of professional responsibilities. For the high moral sensitivity group—which already demonstrated strong ethical awareness—interventions should aim to prevent moral distress and burnout. Regular ethics debriefing sessions, peer support groups, and access to ethics consultation services are recommended, as these nurses may be more prone to emotional exhaustion from over-identification with ethical conflicts. Additionally, the finding that moral sensitivity may decline after 10 years of experience suggests the need for “booster” ethics training and moral resilience programs for mid- to late-career nurses.

At the organizational level, nursing administrators should prioritize role clarity as a modifiable target for intervention. Clear job descriptions, structured orientation programs for new hires, regular performance feedback, and opportunities for role negotiation may enhance role clarity and, consequently, moral sensitivity. Furthermore, creating a supportive ethical climate where nurses feel safe to raise moral concerns without fear of retribution is essential for sustaining high moral sensitivity.

### 4.5 Limitations and future directions

Several methodological limitations should be considered when interpreting the findings of this study. These limitations pertain to the study design, sampling strategy, sample composition, measurement methods, and analytical approach. Each is discussed below with corresponding recommendations for future research. This study has several limitations that should be acknowledged. First, the use of convenience sampling from only one province in China limits the generalizability of our findings. OR nurses in other regions, hospitals of different tiers, or different healthcare systems may exhibit different moral sensitivity profiles. Second, the cross-sectional design precludes causal inferences regarding the relationships between role clarity, demographic factors, and moral sensitivity. Third, the wide confidence interval for the OR of gender suggests a relatively unstable estimate, likely due to the small number of male nurses in our sample. This limits our ability to draw firm conclusions about gender differences in moral sensitivity. Fourth, all data were self-reported, raising the possibility of common method bias and social desirability bias. Although we assured participants of anonymity, nurses with higher moral sensitivity may have been more motivated to participate, potentially introducing selection bias. Fifth, several potentially important variables that may influence moral sensitivity—such as burnout, moral distress, ethical climate, professional identity, and job satisfaction—were not included in our analysis due to questionnaire length constraints and the cross-sectional design. These factors may act as confounders or mediators in the relationship between demographic/occupational characteristics and moral sensitivity. Future studies should incorporate these variables to provide a more comprehensive understanding of the mechanisms underlying moral sensitivity among OR nurses. Finally, our LPA identified two profiles, but the non-significant LMR for the 5-class model and the small class proportions in the 3- and 4-class models suggest that additional profiles may exist in larger or more diverse samples. To address these limitations, future research should prioritize: (a) multicenter stratified sampling to enhance representativeness; (b) longitudinal designs to establish temporal relationships between role clarity and moral sensitivity; (c) larger and more gender-balanced samples to obtain stable estimates of gender effects; (d) incorporation of objective measures or supervisor ratings to complement self-reported data; and (e) validation of the two-profile solution in independent samples across diverse healthcare settings.

## 5 Conclusion

This study is the first to apply LPA to identify two distinct subgroups of moral sensitivity among operating room nurses in China: a low sensitivity group and a high sensitivity group. The overall level of moral sensitivity was moderate. Gender, age, years of service, and role clarity were significant independent associated factors of profile membership. Notably, nurses with 5–10 years of experience showed the highest odds of high moral sensitivity, while those with >10 years showed somewhat lower odds—a finding that warrants further investigation. Role clarity emerged as a modifiable protective factor, suggesting that organizational interventions to clarify job responsibilities may enhance moral sensitivity. Based on these findings, we recommend that nursing managers implement targeted, profile-specific interventions: structured ethics training for the low sensitivity group (younger, less experienced, female nurses) and moral distress prevention programs for the high sensitivity group. Special attention should be paid to mid- and late-career nurses who may be at risk of moral desensitization. At the organizational level, enhancing role clarity through clear job descriptions, structured orientation, and regular feedback may serve as an effective strategy to improve moral sensitivity.

## Data Availability

The raw data supporting the conclusions of this article will be made available by the authors, without undue reservation.
